# Complete Genome Sequences of *Neisseria gonorrhoeae* with Coresistance to First-Line Antimicrobials

**DOI:** 10.1128/genomeA.00966-16

**Published:** 2016-09-08

**Authors:** Amrita Bharat, Irene Martin, Walter Demczuk, Vanessa Allen, David Haldane, Linda Hoang, Michael R. Mulvey

**Affiliations:** a National Microbiology Laboratory, Public Health Agency of Canada, Winnipeg, Manitoba, Canada; b Public Health Ontario Laboratories, Toronto, Ontario, Canada; c Queen Elizabeth II Health Sciences Centre, Halifax, Nova Scotia, Canada; d British Columbia Centre for Disease Control, Provincial Health Authority Services, Vancouver, British Columbia, Canada

## Abstract

*Neisseria gonorrhoeae* strains with coresistance to the first-line antimicrobial treatments azithromycin and ceftriaxone are an emerging public health threat. Here, we present the complete genome sequences of three strains of *N. gonorrhoeae*, including one susceptible strain and two strains with coresistance to ceftriaxone and azithromycin.

## GENOME ANNOUNCEMENT

The World Health Organization and the U.S. Centers for Disease Control have both identified antimicrobial-resistant *Neisseria gonorrhoeae* as an urgent threat to public health (1, 2). *N. gonorrhoeae* has acquired resistance to every previous class of drug that has been approved for treatment, including penicillin (PEN), tetracycline (TET), erythromycin (ERY), and ciprofloxacin (CIP) (3). The current recommended first-line treatment for gonorrhea in the United States, Canada, and Europe is cotreatment with azithromycin (AZM) and one extended-spectrum cephalosporin (ESC), either ceftriaxone (CRO) or cefixime (CFM); however, resistance to each drug, coresistance to both drugs, and treatment failures are increasing (3).

To aid in genome-based surveillance of emerging coresistance to AZM and CRO, we present here the closed genome sequences of three clinical isolates of *N. gonorrhoeae*, including one susceptible to all antimicrobials tested in Canadian surveillance and two with coresistance to AZM and CRO. Coresistance was defined as resistance to AZM (MIC ≥2 µg/ml) (4) and decreased susceptibility to ESCs (MICs in µg/ml of ≥0.125 CRO or ≥0.25 CFM, according to WHO guidelines) (5). Treatment failures have been associated with ESC MICs as low as 0.03 µg/ml (6). NML 34769 (source: cervix of an 18-year-old female in 2011; *N. gonorrhoeae* multiantigen sequence type [NG-MAST] ST-6644; nonmosaic XXII *penA*) was susceptible to all antibiotics tested (MICs in µg/ml of 0.125 AZM, 0.004 CRO, 0.002 CFM, 0.25 PEN, 0.25 TET, 0.5 ERY, 0.004 CIP). NML 34530 (source: urethra of a 42-year-old male in 2012; ST-1407, mosaic XXXIV *penA*) was resistant to all antibiotics tested (MICs in µg/ml of 2 AZM, 0.125 CRO, 0.25 CFM, 4 PEN, 4 TET, 8 ERY, 16 CIP). NML 32867 (source: unknown site of a 34 year-old male in 2010; ST-4980; nonmosaic XII *penA*) was also resistant to all antibiotics tested, including high-level resistance to AZM (MICs in µg/ml of ≥256 AZM, 0.125 CRO, 0.063 CFM, 4 PEN, 4 TET, ≥64 ERY, 32 CIP).

The isolates were sequenced by single-molecule real-time sequencing (SMRT) on the Pacific Biosciences platform by Genome Quebec using one SMRT cell each for NML 34769 (89× coverage), NML 34530 (69× coverage), and NML 32867 (49× coverage). Genomes were assembled with SMRT Analysis software using the hierarchical genome assembly protocol (HGAP) version 3.0, which includes genome polishing with Quiver (7), and were further polished using the RS Resequencing version 1 protocol. Genomes were circularized with Circlator version 1.1.1 (8).

All genomes had 52.4% G+C content, and genome lengths were 2,220,340 bp for NML 34769, 2,228,373 bp for NML 34530, and 2,218,818 bp for NML 32867. The NCBI Prokaryotic Genome Annotation Pipeline (PGAP) (9) annotated four copies of the rRNA operon and 55 tRNAs in each strain, as well as 2,331 coding sequences (CDSs) in NML 34769, 2,338 CDSs in NML 34530, and 2,335 CDSs in NML 32867. NML 34530, which had low-level resistance to azithromycin, contained two copies of the C2611T 23S rRNA (*Escherichia coli* numbering), while NML 32867, which had high-level resistance to azithromycin, contained four copies of the A2059G 23S rRNA.

### Accession number(s).

The complete genome sequences of these *N. gonorrhoeae* strains were deposited in GenBank under accession numbers CP016017 for NML 34769, CP016016 for NML 34530, and CP016015 for NML 32867.
